# Challenges and practical recommendations for successfully recruiting inactive, statin-free older adults to clinical trials

**DOI:** 10.1186/s13104-020-05017-1

**Published:** 2020-03-24

**Authors:** Colleen S. Deane, Bethan E. Phillips, Kenneth Smith, Anna M. Steele, Tina Libretto, Sarah A. Statton, Philip J. Atherton, Timothy Etheridge

**Affiliations:** 1 Department of Sport and Health Sciences, College of Life and Environmental Sciences, University of Exeter, St. Luke’s Campus, Exeter, EX1 2LU UK; 2 Living Systems Institute, University of Exeter, Stocker Road, Exeter, EX4 4QD UK; 3 MRC-ARUK Centre for Musculoskeletal Ageing Research and National Institute of Health Research, Biomedical Research Centre, Division of Medicine and Graduate Entry Medicine, Royal Derby Hospital Centre, School of Medicine, University of Nottingham, Derby, DE22 3DT UK; 4 National Institute for Health Research Exeter Clinical Research Facility, Research Innovation Learning and Development Building, Royal Devon and Exeter NHS Foundation Trust, Exeter, EX2 5DW UK

**Keywords:** Recruitment, Clinical trials, Older adults, Inactive, Statins

## Abstract

**Objectives:**

To outline the challenges and provide practical recommendations for recruiting inactive, statin-free older adults to facilitate feasible study designs. Data was obtained from a double-blind randomised-controlled clinical trial investigating the effects of acipimox versus placebo on muscle function and metabolism in older (65–75 years), inactive, statin-free males. The initial recruitment target was 20 volunteers within 12 months (November 2016–November 2017).

**Results:**

Recruitment occurred via the Exeter 10,000 database containing 236 ‘eligible’ males, a Facebook campaign reaching > 8000 ≥ 65 years old males, 400 directly-addressed letters to ≥ 66 year old males, > 1500 flyers distributed within the community, > 40 emails to local community groups, 4 recruitment talks, 2 magazine adverts and 1 radio advert. Widespread recruitment efforts reaching > 120,000 people led to the recruitment of 20 volunteers (18 completed the clinical trial) within a 25-month timeframe, highlighting the challenge of the timely recruitment of inactive, statin-free older adults for clinical trials. We recommend recruitment for future clinical trials should take a multi-pronged approach from the outset, prioritising the use of volunteer databases, Facebook campaigns and delivering recruitment talks.

## Introduction

The age-related loss of muscle mass (sarcopenia) and strength (dynapenia) is associated with an increased risk of morbidity [12] and mortality [10], costing an estimated £5.7 billion annually (MRC 2012). Clinical trials (CTs) are therefore crucial in order to understand the mechanisms regulating age-related muscle decline, and to develop efficacious therapeutic interventions for the maintenance of muscle health across the life-course.

Volunteer recruitment remains one of the biggest challenges for CTs [11], with three quarters of all CTs failing to meet recruitment deadlines [25] and/or requiring extensions [15]. This is particularly true for CTs looking to recruit healthy older adults due to a higher prevalence of pre-existing diseases [16] and polypharmacy (concurrent use of multiple medications) [13], meaning that the recruitment criteria are often not met. This recruitment challenge is further exacerbated when rigorous study-specific volunteer inclusion/exclusion criteria are applied, such as “physically inactive”, which is often seen as an essential control for robust studies of muscle function and metabolism. In addition, studies investigating the mechanistic basis of drug-based interventions often require invasive tissue sampling (e.g. blood and/or muscle biopsies), both of which may deter older adults from volunteering [16, 22].

It is therefore prudent to disseminate the challenges and suggest practical recommendations for recruiting older adults to CTs that include comprehensive inclusion/exclusion criteria so that an effective recruitment pipeline can be established amongst the ageing research community [21]. This paper reports the successes and challenges associated with the recruitment of 20 healthy older (65–75 years), inactive, statin-free males for a double-blind randomised CT involving exercise testing, blood and muscle biopsy samples to investigate the effects of acipimox versus placebo on muscle function and metabolism.

## Main text

### Clinical trial study design

We aimed to recruit 20 healthy older males between 65 and 75 years of age, over a 12-month period commencing November 2016. Volunteers were excluded if they: had a body mass index (BMI) < 19 or > 29 kg/m^2^, were taking chronic medication known to affect muscle metabolism such as non-steroidal anti-inflammatory drugs (NSAIDs), were smokers, had active/past medical history (PMH) of cardiovascular, respiratory or metabolic disease, renal impairment, musculoskeletal injury, a peptic ulcer, hypersensitivity to acipimox and/or vertigo. To control for activity status, a major confounder in exercise intervention trials, we excluded physically active individuals and thus required inactive volunteers, which we defined as performing no regular planned exercise, for at least the previous 12 months [8]. Finally, the study intervention drug, acipimox, may increase the risk of myopathy when administered concomitantly with a statin [9] (a commonly prescribed lipid-lowering HMG-CoA reductase inhibitor [19]). We therefore excluded individuals taking statins. This study involved five visits in total, each varying in length: (i) an eligibility screening visit (1 h), (ii) an exercise familiarisation session (2 h), and (iii–v) three experimental visits (5 h each). Across the study, each volunteer provided a total of 16 saliva samples, 22 blood samples and 3 muscle biopsy samples (Bergström needle technique [2]). Each volunteer also completed 4 maximal cardiorespiratory fitness tests (VO_2_ max [6]), 4 muscle power tests (Wingate [14]) and 4 physical function assessments (balance, gait speed and chair-rise ability [3]).

### Recruitment methods and success rates

**Fig. 1:**
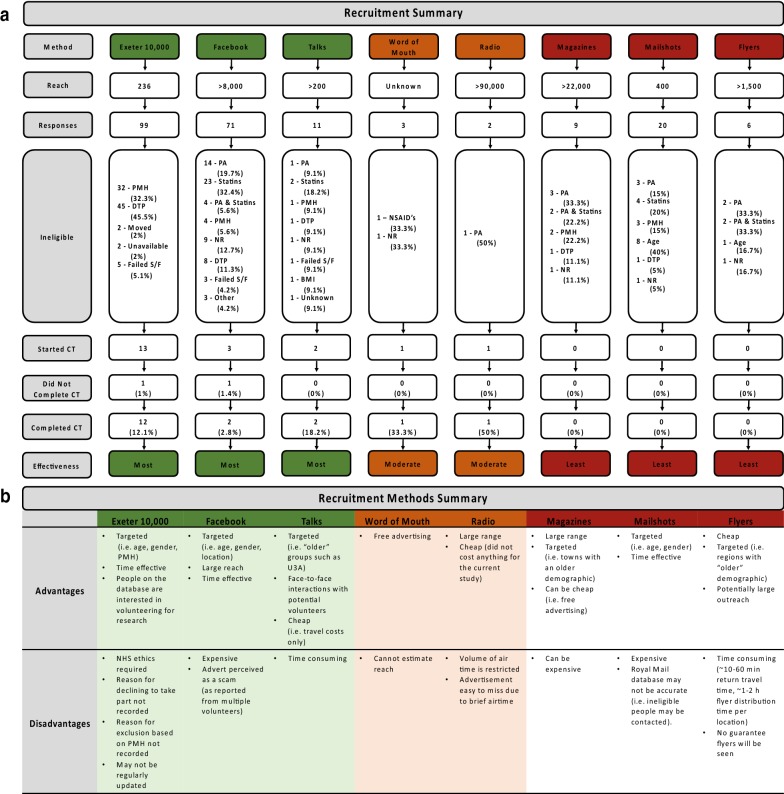
Summary of volunteer recruitment to the CT with each recruitment method (**a**) and the advantages and disadvantages of each recruitment method (**b**). Recruitment methods have been ranked as; most effective (green), moderately effective (amber), least effective (red), based on the number of volunteers recruited to the CT. % represents the proportion of responding volunteers.* BMI* body mass index,* DTP* declined to take part,* NHS* National Health Service,* NR* no response,* NSAIDS* nonsteroidal anti-inflammatory drugs,* PA* physical activity,* PMH* past medical history

Potential volunteers were initially identified from the Exeter 10,000 database, which at the time of conducting searches (November 2016–September 2018) contained demographic and biochemical data from 9763 volunteers that were aged > 18 and living at a permanent address within 25 miles of Exeter (National Institute for Health Research Exeter Clinical Research Facility). Searching the database against the inclusion/exclusion criteria revealed 236 as “eligible”, of which a total of 12 completed the CT (Fig. 1a). As 20 eligible volunteers were not identified through the Exeter 10,000 database, we employed additional recruitment methods that focused on increasing awareness of the CT amongst the general public. This recruitment drive took a multi-pronged approach to maximise reach to our target population and included: a Facebook campaign, demographically targeted (age and gender) postal invitations via the Royal Mail, flyers, presentations at local community groups, adverts in local magazines and local radio segments (Fig. 1a, b).

The Facebook campaign ran four times over a period of 7 months (Additional file 1: Figure S1). The target audience specified in this campaign were: (i) ≥ 65 years old, (ii) male and (iii) living within 20 miles of Exeter. Having reached a minimum of > 8000 individuals within the target audience, a total of 71 responses were recorded with 68 being excluded, 1 not completing the CT and 2 completing the CT (Table 1, Fig. 1a). Thus, this form of social media was an effective method to reach large numbers of the target population, which did lead to the successful recruitment of eligible volunteers (Fig. 1b), although the majority were deemed ineligible due to the strict inclusion/exclusion criteria (i.e. physical activity levels/statins).

**Table 1:** Breakdown of volunteer responses, ineligibility and recruitment rates to each Facebook campaign

Month of campaign	Length of campaign (days)	Reach	Impressions	Frequency	Responses	Ineligible	Reason		Did not complete CT
Jan 18	29	3981	9019	2.27	28	28	9 (32.1%)	Physical activity	0
							7 (25%)	Statins	
							3 (10.7%)	Physical activity and statins	
							4 (14.3%)	PMH	
							2 (7.1)	No response	
							2 (7.1%)	Declined to take part	
							1 (3.6%)	Failed screening/familiarisation	
Feb 18	26	5984	13,651	2.28	13	13	2 (15.4%)	Physical activity	0
							7 (53.9%)	Statins	
							1 (7.7%)	Physical activity and statins	
							1 (7.7%)	Other	
							2 (15.6%)	Failed screening/familiarisation	
Apr 18	26	6740	15,046	2.23	13	11	2 (15.4%)	Physical activity	1 (7.7%)
							2 (15.4%)	Statins	
							2 (15.4%)	No response	
							4 (30.8%)	Declined to take part	
							1 (7.7%)	Other	
Jul 18	4	8244	8418	1.02	17	16	1 (5.9%)	Physical activity	0
							7 (41.2%)	Statins	
							5 (29.4%)	No response	
							2 (11.8%)	Declined to take part	
							1 (5.9%)	Other	

Reach: number of people who saw the advert at least once during a single campaign (i.e. not cumulative over the campaigns). Impressions: the number of times that the advert was on screen. Frequency: the number of times that each person saw the advert

Mailshots (400 to ≥ 66-year-old males, Additional file 1: Figure S2), flyers (> 1500 within the local area) and magazine adverts (2 with a combined reach of > 22,000 homes) led to zero recruits and were thus deemed ineffective methods of recruitment. Personal email contact was made with 43 local community groups (e.g., local Bowls Club and University of the 3^rd^ Age), of which 4 (17.4%) allowed researchers to present a short talk about the research project. From these 4 talks (to > 200 > 65-year-old males), 11 potential volunteers made contact with 2 completing the CT (Fig. 1a). Of the 2 volunteers from this route to complete the CT, 1 recommended a friend, who completed the CT. An additional 2 interested volunteers reported hearing about the study through word of mouth, however 1 was ruled out due to NSAIDs therapy and 1 did not respond to further communications. Thus, targeted talks and word-of-mouth were effective recruitment methods (Fig. 1b). A single radio segment mentioning the CT generated interest from 2 potential volunteers, 1 volunteer completed the CT, the other was excluded on the grounds of being too physically active.

**Fig. 2:**
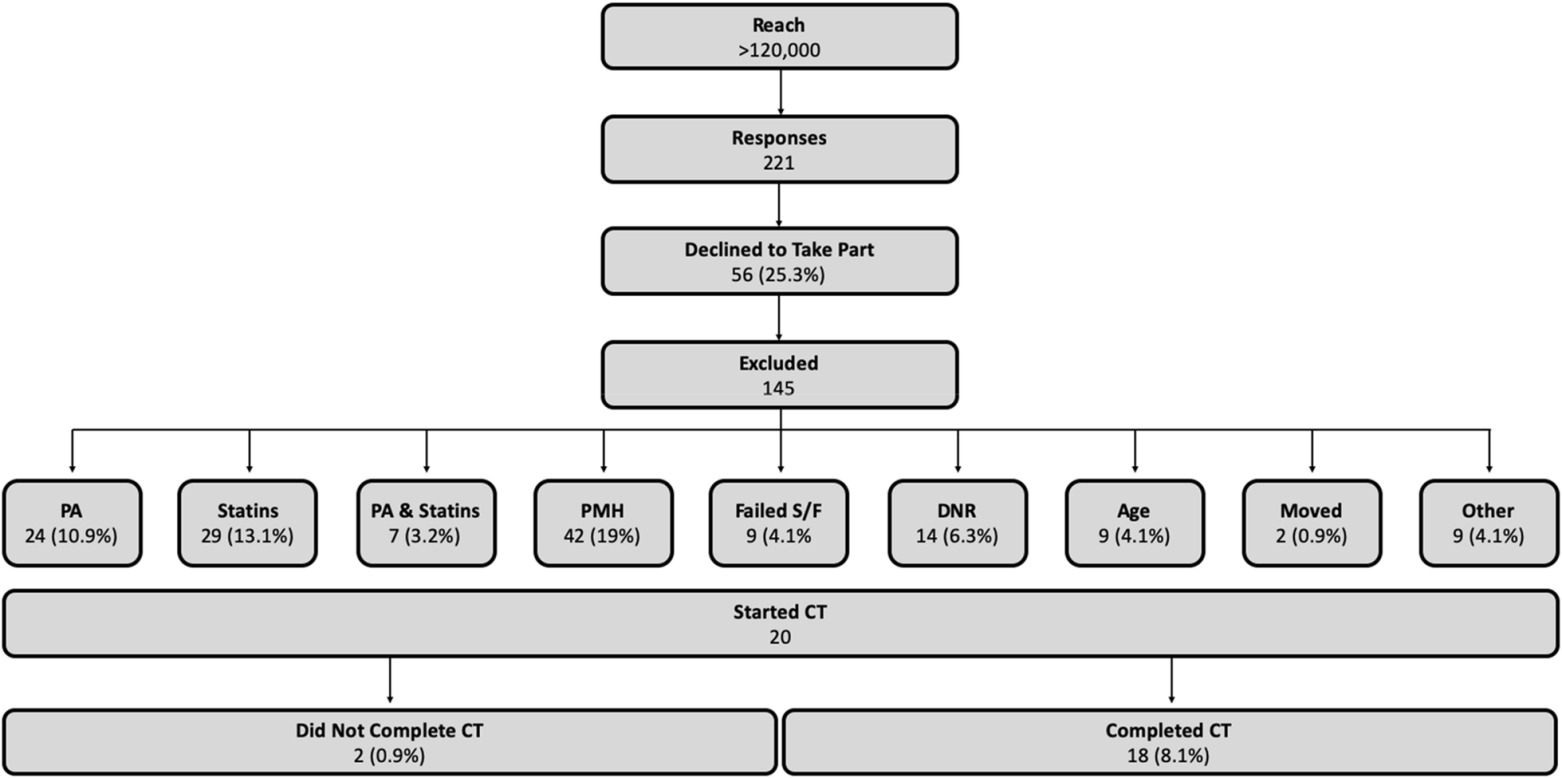
Study recruitment flow chart.* DNR* did not respond, *PA* physical activity,* PMH* past medical history,* S/F* screening/familiarisation

To summarise, we approximate that our recruitment efforts combined reached > 120,000 people, generating 221 responses (Fig. 2). Of these, 18 (8.1%) volunteers completed the CT between November 2016 and December 2018 of which 12 (66.7%) were recruited from the Exeter 10,000 database, 2 (11.1%) from Facebook, 2 (11.1%) from talks at local community groups, 1 (5.6%) from word of mouth and 1 (5.6%) from a radio advertisement. Costs associated with each recruitment method can be found in Additional file 1: Table S1.

## Discussion

This data shows that despite the use of multiple recruitment methods that reached > 120,000 people and elicited 221 responses from interested volunteers, the initial recruitment target of 20 within a 12-month period was not met. Instead, recruitment took 25 months, highlighting the challenge of the timely recruitment of inactive, statin-free older adults to a randomised controlled CT.

Major barriers to recruitment herein were PMH, being too physically active and/or taking statins. This is not surprising as ageing per se increases the risk of cardiovascular-related disease [17], with almost all males over 60 years of age qualifying for statin prescriptions under the National Institute for Healthcare Excellence (NICE) guidelines [24] and physical activity recommended as the most effective countermeasure [20, 23]. This observation highlights a key consideration in that our desired research volunteer population may not be representative of the ageing population. As such, follow-on studies, in which elucidating the underpinning mechanisms of the intervention is not the primary aim, are needed in a more representative ageing population (e.g. recreationally active).

Although more lenient inclusion/exclusion criteria would likely result in substantially higher recruitment rates [26], for many CTs this is not possible due to serious safety concerns (e.g. relative contraindications) and potential impact on scientific rigour (e.g. introduction of major confounders into the study design). For example, in the current study statin prescription was excluded due to possible contraindications with the acipimox drug intervention, where simultaneous consumption could lead to muscle toxicity [9]. Further, endurance and/or resistance exercise training improves muscle strength, muscle mass, mitochondrial metabolism and exercise capacity [7], so for this study it was critical for scientific robustness that inactive volunteers were recruited.

### Practical recommendations

Of all of the recruitment methods used herein, the Exeter 10,000 database provided the largest number of volunteers to complete the CT (Fig. 1a). However, searches of the database initially identified 236 people as “eligible”, where in actual fact > 30% of these were ineligible due to PMH. This suggests that the detail within the database (i.e. records of medications/PMH) may not be accurate, perhaps due to changes in volunteers’ characteristics, which were not updated (Fig. 1b). Nonetheless, where possible we recommend that volunteer databases form the foundations of recruitment drives for future CTs. However, the awareness and utility of such databases among CT teams is likely low since there is not a central resource (i.e. website) detailing the recruitment databases available within the UK. Further, access to these databases may be restricted if they are purpose-built, staffed and/or funded by certain research centres or organisations. Although larger-scale, regional or national databases would likely prove highly valuable for research volunteer recruitment, these may not be feasible due to data protection and ownership/management considerations. We have, however, identified through personal communications with other researchers in the UK (i.e. the University of Nottingham Clinical Physiology research group at the Royal Derby Hospital) that the re-recruitment of research volunteers via an annually updated internal database is an effective recruitment method for ageing research, and we therefore suggest that establishing internal recruitment databases may facilitate successful and timely volunteer recruitment, if these databases are managed and updated appropriately.

Since we were unable to recruit all 20 volunteers through Exeter 10,000, we later employed alternative research strategies within Exeter (16% of population are > 65 years old), but also within surrounding rural areas due to the larger proportion of older adults (23–30% of population are > 65 years old) in these geographical regions (Office of National Statistics, [18]. Effective methods that did lead to volunteer recruitment were Facebook and the radio. Demographically targeted letters, flyers and magazine adverts were all ineffective for this study, similar to previous experience [1].

In summary, we highlight that stringent inclusion/exclusion criteria present significant challenges to volunteer recruitment into CTs. In the absence of a completely effective ‘gold-standard’ recruitment method or pipeline, we provide suggestions for an experience-based framework for optimising recruitment strategies to benefit the wider CT community, and more specifically those involved in ageing research. This framework proposes a multipronged approach that utilises existing volunteer databases combined with targeted social media and community group recruitment approaches undertaken simultaneously from the outset of a CT. We also wish to highlight that despite a growing body of CTs in the UK, a lack of public knowledge about where to find information on recruiting CTs is an additional challenge for recruitment (House of Commons [5], with a central website a potential solution to this.

## Study limitations

- The challenges and successes of recruitment outlined herein are based on only one study conducted in the South West (UK) and it is possible that recruitment may be different based on geographical locations. For example, the West Midlands have a greater percentage of inactive people (compared to the South West) [4] and thus, it may be easier to recruit inactive volunteers within that region.
- The inability to identify overlap between people exposed to each recruitment strategy and the lack of precision in the estimation of reach may impact the effectiveness of each recruitment strategy.
- The recruitment strategies deemed as “effective” or “ineffective” herein may be relative to the small recruitment target (i.e. 20) and thus, may not be wholly suitable for CTs with larger recruitment targets.

## Supplementary information

**Additional file 1: Table S1.** Costs associated with each recruitment method**. Figure S1.** Facebook campaigns**. Figure S2.** Mailshot template.

## Data Availability

The dataset used and/or analysed for the current study are available from the corresponding author on reasonable request.

## Abbreviations

BMI: Body mass index
CT: Clinical trial
DNR: Did not respond
DTP: Declined to take part
ECG: Electrocardiogram
Fam: Familiarisation
NHS: National health service
NR: No response
NSAIDs: Nonsteroidal anti-inflammatory drugs
PA: Physical activity
PIS: Participant information sheet
PMH: Past medical history
S/F: Screening/familiarisation
