# Supplementary material for: Challenges and practical recommendations for successfully recruiting inactive, statin-free older adults to clinical trials
Source: BMC Res Notes. 2020 Mar 24;13:174. doi: 10.1186/s13104-020-05017-1 (PMC7092412; doi:10.1186/s13104-020-05017-1)
Supplement: Supplementary file 1 — Additional file 1: Table S1. Costs associated with each recruitment method. Figure S1. Facebook campaigns. Figure S2. Mailshot template. [file 13104_2020_5017_MOESM1_ESM.docx]

**Table S1. Costs associated with each recruitment method.**

| **Recruitment Method** | **Direct Costs** | **Indirect Cost Considerations** |
| --- | --- | --- |
| **Exeter 10,000** | Costs include database searches, initial contact and associated paperwork    Total = £526.40 | - Staff time to follow up potential volunteers |
| **Facebook** | £100 per campaign  Total = £400 | - Staffing time to follow up potential volunteers |
| **Talks** | Free of charge | - Transportation - Staff time to do the talks - Staff time to follow up potential volunteers |
| **Word of mouth** | Free of charge | - Staff time to follow up potential volunteers |
| **Radio** | Free of charge | - Staff time to do the radio advert - Staff time to follow up potential volunteers |
| **Magazines** | 1 magazine with a reach of 19,952 homes - £235  1 magazine with a reach of >3000 homes - Free of charge  Total = £235 | - Staff time to create magazine advert - Staff time to follow up potential volunteers |
| **Mailshots** | £141.60 per 200 mailshots  Total = £283.20 | - Staff time to create mailshot - Staff time to follow up potential volunteers |
| **Flyers** | ~1500 flyers * £0.065  (cost of printing a single colour sheet)  Total = ~£97.50 | - Transportation - Staff time to create magazine advert - Staff time to follow up potential volunteers |

**Figure S1.** **Facebook campaigns.** The depicted Facebook advert appeared on the news feed of >8000 people within the target audience, which was; ≥65 years old males living within 20 miles of Exeter. The reach of the Facebook campaign refers to the number of people who saw the advert at least once during a single campaign (i.e. not cumulative over the four campaigns). It cannot be guaranteed that there was no overlap of people being targeted between campaigns, thus the overall highest reach of a single campaign (i.e. 8244) was used to suggest the absolute minimum of our target audience that saw the Facebook advert. Those interested in finding out more about participating in the study were able to contact the research team using contact details on the advert (removed for publication purposes). To make the Facebook advert stand out when it appeared on new feeds, a photo of an older male in a gym setting (placed below the text) was included, however, it has been removed from this supplemental due to copyright purposes.


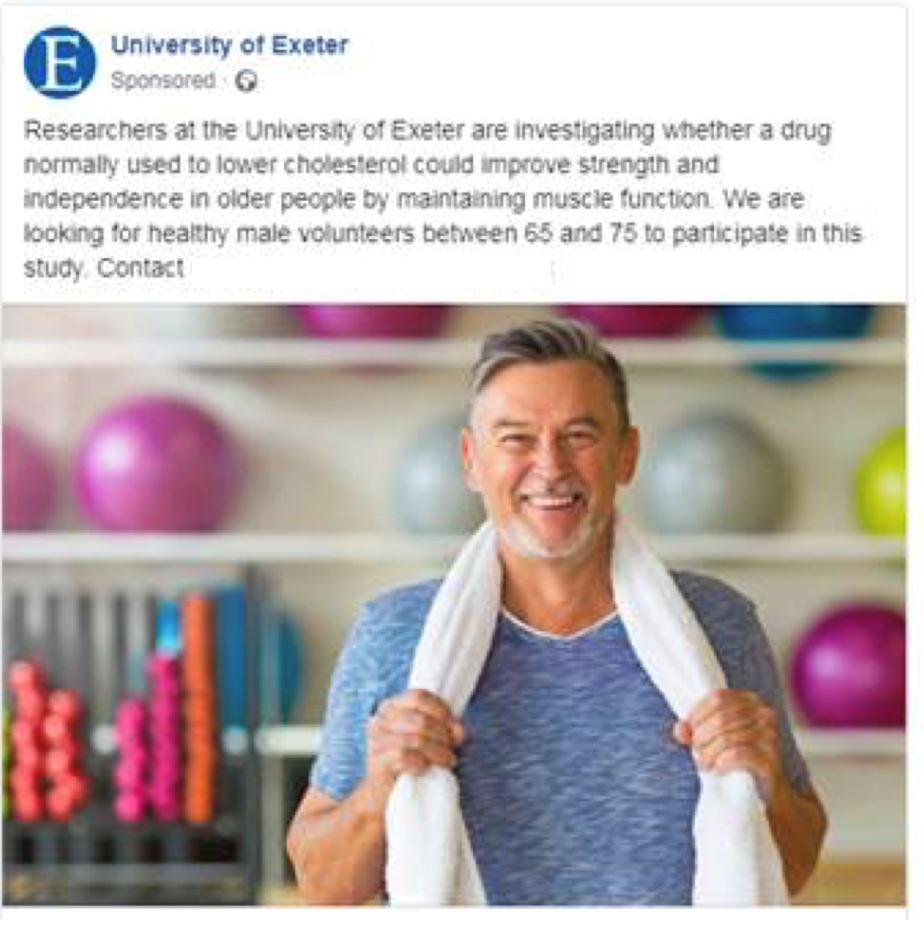


**Figure S2.** **Mailshot template.** Study invitation letters were sent to 400 ≥66-year-old males using the online Royal Mail Mailshot Maker system. Those interested in finding out more about participating in the study were asked to contact the research team using contact details on the letter (removed for publication purposes). ****
